# Influence of Wood Ash on the Mechanical Properties and Durability of Cement Mortars

**DOI:** 10.3390/ma19153186

**Published:** 2026-07-26

**Authors:** Oskars Lescinskis, Genadijs Sahmenko, Girts Bumanis, Diana Bajare

**Affiliations:** Institute of Sustainable Building Materials and Engineering Systems, Faculty of Civil and Mechanical Engineering, Riga Technical University, 6A Kipsalas, LV-1048 Riga, Latvia; genadijs.sahmenko@rtu.lv (G.S.); girts.bumanis@rtu.lv (G.B.)

**Keywords:** wood ash, Portland cement, cement mortar, cement replacement

## Abstract

This study investigates the influence of wood fly ash (WFA) and wood bottom ash (WBA) as a partial replacement of Portland cement (PC) on the mechanical performance and durability of cement mortars. Mortar mixtures containing 20% WFA (FA-20) and 20% WBA (BA-20) were compared with a reference mixture (REF) using bending and compressive strength tests, ultrasonic pulse velocity (UPV), total water absorption (TWA), and durability tests such as alkali–silica reaction (ASR) and carbonation resistance. The results showed that BA-20 exhibited higher mechanical performance and a denser microstructure than FA-20, as confirmed by UPV and TWA. At 365 days, compressive strength reached 71.9 MPa for REF, 64.3 MPa for BA-20, and 43.7 MPa for FA-20. Durability results indicated that after 365 days, REF exhibited the highest ASR expansion (~0.50%), whereas the incorporation of wood ash reduced expansion to approximately 0.41% for FA-20 and 0.29% for BA-20. In terms of carbonation resistance, FA-20 showed the greatest accelerated carbonation depth (10–14 mm), while REF exhibited the lowest carbonation depth (~3 mm). The differences were attributed to PC dilution and microstructural variations affecting porosity and transport properties. WBA demonstrated better performance than WFA, highlighting the importance of wood ash particle characteristics in PC replacement applications.

## 1. Introduction

Portland cement (PC) is one of the most widely used construction materials worldwide; however, its production is associated with significant environmental impacts. The cement industry contributes approximately 7–9% of global anthropogenic CO_2_ emissions, primarily due to the energy-intensive and carbon-intensive clinker production process. In addition to greenhouse gas emissions, cement manufacturing consumes large quantities of natural raw materials, raising concerns regarding long-term resource sustainability [1,2].

To reduce the environmental footprint of cement-based materials, the partial replacement of PC with alternative mineral materials has been widely investigated. Traditionally used industrial by-products such as coal fly ash have been incorporated into cement systems due to their availability and performance benefits [3]. However, the global shift away from coal-based energy production has led to a gradual reduction in the availability of coal fly ash, creating a need for alternative materials suitable for partial replacement of PC [4].

In recent years, increasing attention has been given to ashes derived from biomass combustion as a potential partial replacement for PC. The growing use of wood biomass as a renewable energy source has resulted in significant production of wood ash (WA), which is often considered a waste product requiring appropriate management [5]. Depending on the combustion process and fuel type, WA may vary widely in chemical composition and physical properties [6]. WA, including wood fly ash (WFA) and wood bottom ash (WBA), are among the most generated residues in biomass-fired boilers and therefore represent a relevant material stream for potential reuse in cement-based systems [7].

WFA and WBA differ mainly in their formation conditions and particle size distribution [8,9]. WFA is typically finer and more easily dispersed, while WBA is generally coarser and more heterogeneous [10]. These physical differences may influence their behavior when incorporated into cementitious matrices, particularly in terms of packing density, water demand, and mechanical performance of hardened materials [11].

Previous studies have reported that WA can be used as partial replacement material of PC in cement mortars and concrete. However, the resulting mechanical performance is strongly dependent on the type of WA (fly ash or bottom ash), particle characteristics, and replacement level. In many cases, increasing WA content leads to a reduction in compressive strength, although acceptable performance has been observed at lower replacement levels depending on material properties [9,12,13].

Although numerous studies have investigated the use of WA as partial replacement of PC, direct comparisons between WFA and WBA originating from the same biomass combustion source remain limited. Furthermore, the long-term performance of cement-based materials containing these ashes has received comparatively little attention, with most studies focusing on short- and medium-term curing periods. Therefore, the present study investigates the direct use of WFA and WBA, derived from the same biomass combustion source, as materials for partial replacement of PC in cement mortar mixtures. The ashes were characterized using X-ray fluorescence (XRF), X-ray diffraction (XRD), particle size distribution analysis, and scanning electron microscopy (SEM). The evolution of the cementitious systems was monitored during the first 72 h using ultrasonic pulse velocity (UPV) continuous measurements, while the long-term performance was evaluated through compressive strength, water absorption, and UPV measurements after 7, 28, 90, and 365 days of curing. In addition, the durability-related performance of the mortars was assessed through alkali–silica reaction (ASR) and carbonation tests, the latter being performed on specimens cured for 365 days. The study provides insight into the influence of WA type on both the early-age development and long-term performance of mortar, contributing to the evaluation of wood combustion residues as alternative constituents in more sustainable cement-based materials.

## 2. Materials and Methods

### 2.1. Wood Ash Characterization

**Figure 1:**
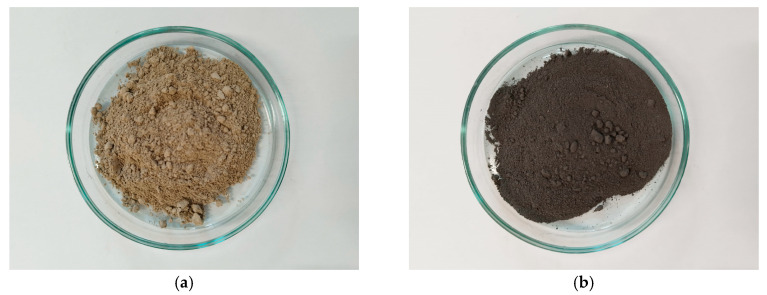
(**a**) WFA and (**b**) WBA in Petri dishes of a diameter of 10 cm.

The wood ash (WA) used in the study was collected from the thermal plant Liepajas Energija Ltd. In Liepāja, Latvia. Two WA fractions—wood fly ash (WFA) and wood bottom ash (WBA) were collected. To reduce the particle size of WBA and make it more homogeneous, it was first sieved through a 2 mm sieve to remove the larger unburnt carbon particles and subsequently ball-milled at 300 rpm for 5 min by using a Retsch Planetary Ball Mill PM 400 (Retsch GmbH, Haan, Germany). This ground fraction was then used throughout the study. In this study, WFA was not subjected to ball-milling. WFA and WBA samples are shown in Figure 1.

The chemical compositions of WAs were determined using an X-ray fluorescence (XRF) spectrometer Rigaku Supermini200 (Rigaku Electric Co., Ltd., Tokyo, Japan) equipped with a 200 W Pd X-ray tube. Measurements were performed in a helium atmosphere.

The crystalline phases of WAs were identified by using an X-ray diffractometer (XRD) Aeris Panalytical (Malvern Panalytical B.V., Almelo, The Netherlands) using the CuKα radiation at a generator voltage 40 kV and tube current 15 mA with the wavelength of 1.5406 Å, the 2θ scanning range of 10 to 70°, the step size of 0.02° and the speed of scanning of 3°/min.

The structure, size and shape of WAs were investigated using a scanning electron microscope (SEM) Thermo Scientific Verios 5 UC (Thermo Fisher Scientific, Waltham, MA, USA). Before the SEM analysis, the WA samples were coated with a 10 nm layer of carbon by using a LEICA EM ACE 200 coater (Leica Microsystems, Wetzlar, Germany).

The particle size distributions of WAs were determined by using the wet sieving method according to the LVS EN 933-1 standard [14]. Prior to testing, both WA samples were oven-dried at 110 °C until constant mass was achieved to remove residual moisture. Then, 200 g of each dried sample was weighed and subjected to wet sieving through a series of standard sieves arranged in descending mesh size (largest opening at the top, smallest at the bottom). The sieving procedure was performed manually using a standard sieve stack and continuous water jet to facilitate particle dispersion and prevent agglomeration. The material passing through the finest sieve was collected in a receiving pan together with the water flow, ensuring full recovery of fine particles. After sieving, the material retained on each sieve was collected, dried, and weighed. The cumulative percentage passing was then calculated to determine the particle size distribution of the WAs.

### 2.2. Raw Materials and Mixture Designs

A locally manufactured PC, Schwenk (Brocēni, Latvia) CEM I 52.5 N, was used in this study. The cement contains more than 95% clinker and complies with the requirements of LVS EN 197-1:2012 [15]. The chemical composition of the PC is given in Table 1. Sand was obtained from the local manufacturer Saulkalne S Ltd. (Saulkalne, Latvia), with the grain sizes of 0–2 mm and 0.6–2.5 mm used at proportions of 80% and 20%, respectively. The liquid superplasticizer (SP) Mapei was used to improve workability and maintain the slump flow of the fresh mortars within the limits of 160–170 mm, according to the procedure described in LVS EN 1015-3:2000 [16].

The mixture compositions of mortar samples and their designations are given in Table 2. In total, 3 mortar mixtures were prepared: (1) reference mixture REF without WA, (2) mixture containing WFA, and (3) mixture containing WBA. WAs were incorporated as a 20% replacement of PC by mass. A 20% cement replacement level was selected as a representative intermediate dosage within the commonly investigated range for biomass and wood-derived ashes (approximately 10–20%), for which several studies report acceptable mechanical and durability performance [2,18]. In all mixtures, the combined mass of PC and WA was used as the basis for mixture proportion calculations. All mixture proportions are expressed per 1000 g of PC + WA. The water-to-(PC + WA) and sand-to-(PC + WA) ratios were kept constant at 0.45 and 2.8, respectively. A slump flow of 160–170 mm was achieved by adjusting the dosage of superplasticizer (SP). The use of SP ensured comparable fresh-state properties among all mixtures, allowing evaluation of the influence of WA incorporation on mortar behavior while maintaining a constant water-to-(PC + WA) ratio.

### 2.3. Mortar Properties

#### 2.3.1. Fresh and Early-Age Mortar Behavior

Ultrasonic pulse velocity (UPV) measurements were carried out using an UltraTest GmbH IP-8 system (UltraTest GmbH, Achim, Germany) with a fixed transmitter–receiver configuration and a transducer spacing of 40 mm. Fresh cementitious mixtures were poured into molds and lightly compacted to promote uniform distribution and reduce entrapped air. The molds were then sealed with plastic film to prevent moisture loss during early-age UPV monitoring. Continuous UPV monitoring commenced immediately after pouring, with ultrasonic signals recorded at 1 min intervals for 72 h to evaluate early-age hydration behavior and microstructural evolution in cementitious systems.

#### 2.3.2. Compressive and Bending Strengths

**Figure 2:**
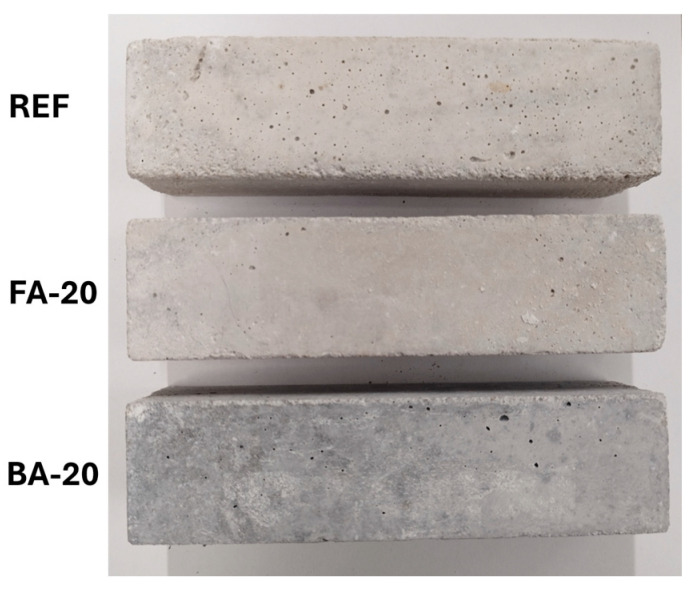
Hardened mortar prism specimens (REF, FA-20, and BA-20) used for mechanical testing.

Compressive and bending strengths for all mortar samples were determined according to the LVS EN 196-1 standard [19]. Mortar specimens were produced in 40 mm × 40 mm × 160 mm steel molds. The mortar specimens were removed from the molds 24 h after production and placed in water at room temperature. Strength tests were carried out after 7, 28, 90 and 365 days. A three-point bending test was performed with a span of 100 mm and a test speed of 0.5 mm/min by using a WDW-20 20 kN testing machine (Jinan Hensgrand Instrument Co., Ltd., Jinan, China). A compressive test on 62.5 mm × 40 mm × 40 mm mortar specimens was performed with a test speed of 0.8 MPa/s by using a Controls 3000 kN compression machine (Controls S.p.A., Milan, Italy). The hardened mortar specimens of REF, FA-20, and BA-20 used for mechanical testing are shown in Figure 2.

#### 2.3.3. UPV of Hardened Mortar Specimens

The UPV for hardened mortar specimens was determined after 7, 28, 90 and 365 days using a UK1401 ultrasonic tester (ACS-Solutions GmbH, Saarbrücken, Germany). These measurements were carried out on 40 mm × 40 mm × 160 mm specimens. Before the test, specimens were removed from water and dried with a cloth. Each side or the prism was measured 3 times and in total 3 parallel samples were used for each mixture composition, and the average value was calculated.

#### 2.3.4. Total Water Absorption

The total water absorption (TWA) was determined after 7, 28, 90 and 365 days according to ASTM C642-21 [20]. Hardened mortar specimens were dried to constant mass at 105 °C for 48 h. After drying, the specimens were cooled to room temperature in dissector and weighed. Subsequently the specimens were immersed in water at room temperature for 72 h, after which they were removed, surface-dried, and weighed. The TWA was calculated according to Equation (1):

(1) $$TWA\left(\%\right)=\frac{m_{sat}-m_{dry}}{m_{dry}}\times 100$$

where:

$m_{sat}$—mass of a dried specimen saturated in water for 72 h;

$m_{dry}$—mass of a specimen dried at 105 °C for 48 h.

#### 2.3.5. Alkali-Silica Reaction Test

The alkali–silica reaction (ASR) behavior of the mortar specimens was evaluated in accordance with the RILEM AAR-2 Accelerated Mortar Bar Test [21], using 40 mm × 40 mm × 160 mm prisms prepared as described previously. After demolding, the specimens were immersed in water at 80 °C for 24 h to ensure sufficient hydration and to promote an accelerated reaction (conditioning). After removal from the water, the prisms were measured, and their lengths were recorded as the initial reference values for comparison. The specimens were subsequently transferred to a 1 M NaOH solution maintained at 80 °C, where they were stored for an extended period of up to 1 year. Length change measurements at the very initial stage (up to 80 days) were taken at regular intervals to monitor expansion. The ASR expansion was calculated according to Equation (2):

(2) $$ASR\;expansion\left(\%\right)=\frac{L_{X}-L_{0}}{160}\times 100$$

where:

$L_{X}$—length (mm) of a prism exposed to 1 M NaOH at 80 °C for a specified period;

$L_{0}$—length (mm) of a prism after immersion in water at 80 °C for 24 h.

The measured expansion was used to evaluate the influence of WA incorporation on ASR-related behavior. The extended testing period was adopted to observe the long-term expansion trends and to better assess the effectiveness of WA in mitigating ASR beyond the standard test duration of 14 days.

#### 2.3.6. Accelerated Carbonation Tests

A modified accelerated carbonation test based on EN 12390-12 [22] was performed using a CO_2_ concentration of 20% to accelerate carbonation. The test was conducted on 40 mm× 40 mm × 160 mm mortar prism specimens cured for 365 days in a carbonation chamber at the following parameters: CO_2_ concentration of 20%, temperature of 20 °C and the relative humidity of 70% for 14 days. The test was conducted to evaluate the influence of WFA and WBA content on the carbonation depth of mortar specimens. Before the carbonation process, specimens were removed from water and conditioned at room temperature and 60% relative humidity for 48 h. After 14 days of accelerated carbonation, the mortar specimens were split in halves and sprayed with 1% phenolphthalein solution in ethanol. The carbonation depth was then compared with that of non-carbonated mortar specimens stored at room temperature wrapped in plastic film to prevent drying. The CO_2_ concentration applied in the study exceeded conventional standardized conditions (typically 1–5% CO_2_); therefore, the results should be interpreted primarily for comparative evaluation of material performance rather than direct prediction of natural carbonation behavior.

## 3. Results and Discussion

### 3.1. XRF and XRD of WAs

**Figure 3:**
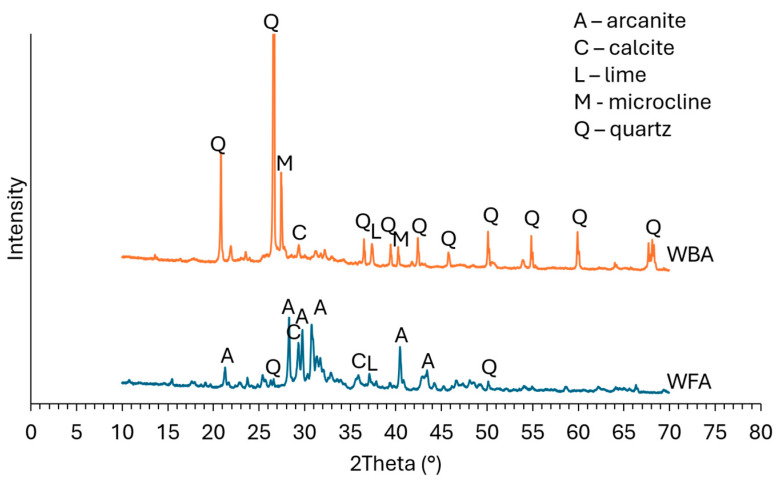
XRD patterns of WFA and WBA.

The XRF results for each WA type are presented in Table 3, while the XRD patterns are shown in Figure 3. For comparison, the chemical composition of PC used in this study is provided in Table 1. In the WFA sample, two dominant crystalline phases—quartz and arcanite—were observed. Quartz was identified as the dominant crystalline phase in the WBA sample along with low-intensity peaks of lime and calcite.

The sum of SiO_2_, Al_2_O_3_, and Fe_2_O_3_ contents is commonly used as an indicator for the assessment of siliceous fly ash according to EN 450-1 [23]. Although this standard was developed for coal-derived fly ash and is not directly applicable to biomass ashes, this parameter can provide a useful basis for comparison. In the present study, the calculated SiO_2_ + Al_2_O_3_ + Fe_2_O_3_ values were 7.0 wt.% for WFA and 57.0 wt.% for WBA, which are lower than the 70 wt.% value commonly associated with siliceous coal fly ash. In addition, both investigated ashes exhibited relatively high CaO contents (26.2 wt.% for WFA and 24.2 wt.% for WBA), together with elevated K_2_O contents, particularly for WFA. These compositional characteristics are consistent with the typical variability of biomass-derived ashes and reflect differences in fuel composition and combustion conditions compared with coal fly ash. Similar observations have been reported in previous studies, where wood biomass ashes were shown to differ from the criteria established for coal fly ash according to EN 450-1, while still being investigated as alternative cement replacement materials [18,24,25]. Therefore, the suitability of WFA and WBA in the present study was evaluated based on their influence on the fresh, mechanical, and durability-related properties of cement mortars.

The chemical compositions of WFA and WBA indicate notable differences that may influence their performance in cementitious systems. Compared with WBA, WFA contained substantially higher contents of SO_3_, K_2_O, Cl^−^, and ZnO. The elevated K_2_O and SO_3_ contents should be considered when evaluating the use of WFA in cement-based materials, as they may influence the potential for alkali–silica reaction and sulfate-related reactions within the cementitious system. In addition, the higher Cl^−^ content should be considered if WFA is intended for reinforced concrete applications because of its potential implications for reinforcement corrosion, while the elevated ZnO content may also affect the hydration behavior of the cementitious system. Although no adverse effects specifically attributable to these constituents were identified under the conditions investigated in the present study, their presence should be considered when assessing the practical application of WFA as a partial replacement for PC.

### 3.2. SEM Results of WAs

**Figure 4:**
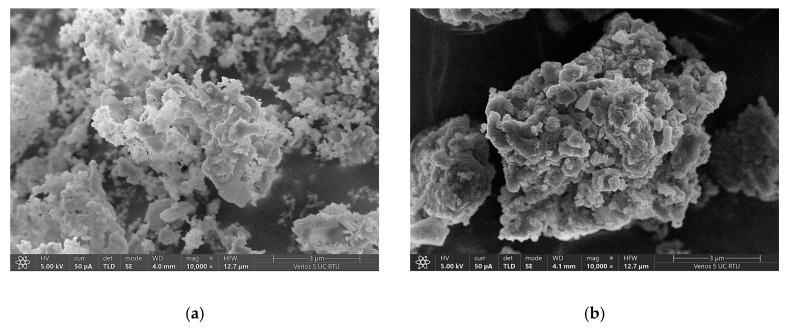
SEM images of (**a**) WFA and (**b**) WBA.

Figure 4 shows the morphological differences between WFA and WBA observed by SEM analysis. The WFA particles appear relatively finer and more loosely agglomerated, with irregular flaky structures and heterogeneous surface textures. In contrast, the WBA exhibits denser and coarser agglomerates with more compact particle arrangements and rougher external surfaces. Both materials present irregular particle geometry, porous features, and angular morphologies characteristic of biomass combustion residues [26,27].

The observed morphological differences are expected to influence the fresh and hardened properties of the mortars. In particular, the finer and more heterogeneous WFA particles may increase water demand and contribute to a more porous microstructure, while the denser and coarser WBA agglomerates may enhance particle packing and lead to a more compact matrix, thereby affecting durability-related performance such as TWA, ASR resistance, and carbonation, while also influencing UPV, which reflects the development of microstructural densification.

### 3.3. Particle Size Distribution of Was

**Figure 5:**
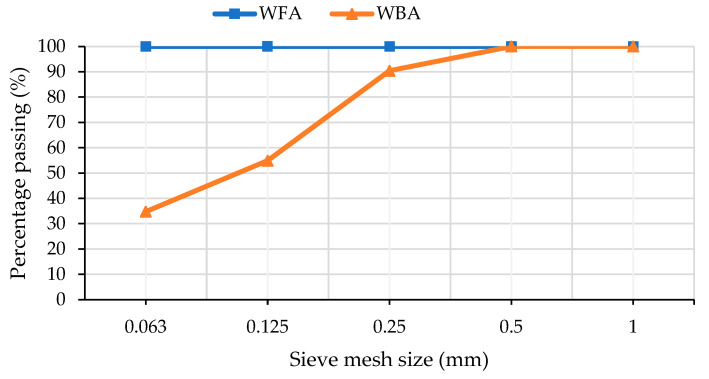
Particle size distribution of WFA and WBA.

The obtained particle size distribution curves determined by the wet sieving method are presented in Figure 5. The results indicate that WFA, analyzed in its as-received condition, consisted predominantly of very fine particles, with 100% passing through all sieve sizes. In contrast, WBA, which was ball-milled for 5 min at 300 rpm, exhibited a broader and coarser particle size distribution, with approximately 35%, 55%, and 90% passing through the 0.063, 0.125, and 0.25 mm sieves, respectively, while complete passage was achieved on the 0.5 mm sieve. These findings demonstrate that WFA was significantly finer and more uniform compared with WBA.

### 3.4. Fresh and Early-Age Mortar Behavior

**Figure 6:**
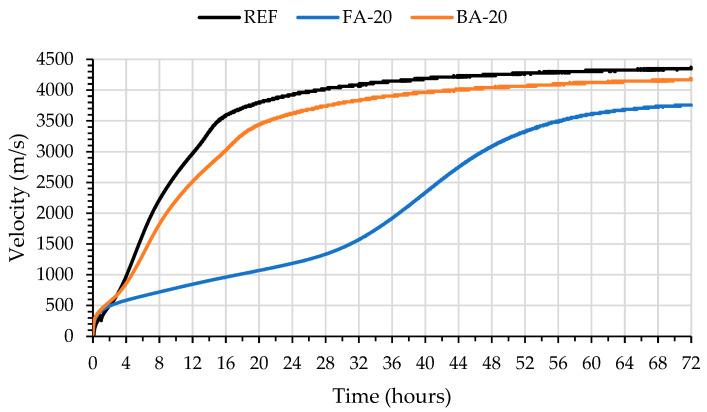
UPV development and comparison of REF, FA-20 and BA-20 mixtures.

The evolution of UPV with time, shown in Figure 6, reflects the progressive development of internal structure in the mortars during early-age hydration. The REF, composed of PC, exhibited the most rapid increase in UPV, with a steep rise within the first 16 h, indicating rapid setting and early formation of a continuous hydration product network. The curve reached a plateau at approximately 4200–4300 m/s after ~36 h, suggesting the development of a dense and well-connected microstructure associated with early hydration products.

The BA-20 followed a similar overall trend but with a slightly delayed initial response. The early-stage increase in UPV was more gradual than that of the REF, indicating a moderate retardation of hydration kinetics due to the dilution of PC. However, the system exhibited steady development within the first 20 h and approached a slightly lower final velocity (~4000–4100 m/s) compared to the REF, suggesting that the WBA may contribute to later-age densification mainly through filler effects.

In contrast, the FA-20 showed a noticeably delayed UPV development. The FA-20 mixture required a significantly higher dosage of SP to achieve the target workability compared with the REF and BA-20 mixtures, which is consistent with its finer particle size distribution (Figure 5). This increased SP demand may have influenced early hydration kinetics and microstructural development through enhanced particle dispersion and possible delay in early structure formation. This behavior is consistent with the observed delayed UPV development and lower early-age mechanical performance, suggesting that the higher SP dosage may have contributed, alongside the intrinsic characteristics of WFA, to the initial differences in structural development. The increase in UPV remained gradual up to approximately 30 h, indicating a prolonged induction period and limited early structural development. Although some acceleration occurred after this stage, the final UPV values remained significantly lower (~3600–3800 m/s), implying a less dense and more heterogeneous microstructure compared to the other mixtures.

These UPV trends are consistent with the particle morphology of WFA and WBA observed by SEM analysis (Figure 4). In particular, the FA-20 mixture containing WFA, which exhibited a more heterogeneous morphology with loosely packed and irregularly shaped particles, corresponds to a more gradual UPV development and a lower final velocity, indicating a less compact internal structure. In contrast, the BA-20 mixture containing WBA, characterized by denser agglomerates and a more compact particle arrangement in the SEM image, aligns with faster UPV development and a higher ultimate velocity, suggesting a more continuous and compact microstructure.

### 3.5. Mechanical Performance of Mortar Samples

**Figure 7:**
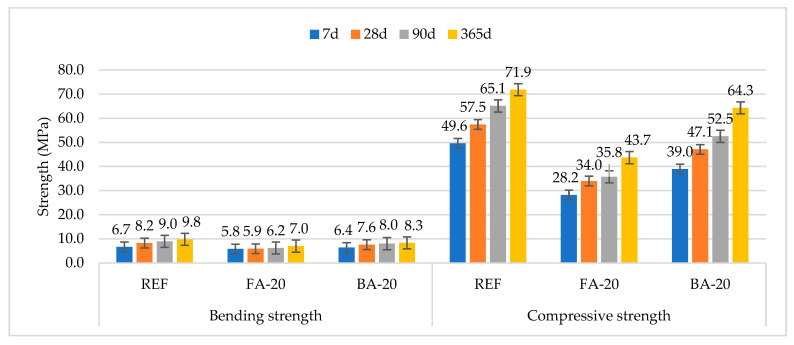
Bending and compressive and strength of hardened mortar specimens.

The mechanical performance of the REF, FA-20, and BA-20 was evaluated through bending and compressive strength tests conducted at curing ages of 7, 28, 90, and 365 days. The obtained results are presented in Figure 7.

The compressive strength results showed a continuous increase with curing time for all mixtures. The REF exhibited the highest compressive strength throughout the entire testing period, increasing from 49.6 Mpa at 7 days to 71.9 Mpa after 365 days. The FA-20 showed the lowest early-age and final strength, reaching 28.2 Mpa at 7 days and 43.7 Mpa at 365 days, respectively. In contrast, the BA-20 exhibited significantly higher early-age strength compared with the FA-20, achieving 39.0 Mpa at 7 days and increasing to 64.3 Mpa at 365 days. The improved performance of WBA can be attributed to a combination of a broader particle size distribution, and improved particle packing, which collectively contributed to a denser microstructure. Improved particle packing can reduce the volume of voids and contribute to the formation of a denser microstructure [28]. It should also be noted that, in addition to intrinsic differences between WFA and WBA, the two materials were subjected to different preprocessing procedures. WBA was sieved and ball-milled prior to use, whereas WFA was used in its as-received condition. Therefore, the observed differences in mechanical performance reflect both the type of WA and particle size/processing effects. This interpretation is consistent with the lower water absorption and higher UPV values observed for the BA-20 compared with the FA-20 [13].

A similar trend was observed in bending strength development. The REF showed the highest performance, increasing from 6.7 Mpa at 7 days to 9.8 Mpa at 365 days. The FA-20 demonstrated the lowest initial bending strength (5.8 Mpa at 7 days), followed by gradual improvement to 7.0 Mpa at 365 days. The BA-20 showed intermediate behavior, with bending strength increasing from 6.4 Mpa at 7 days to 8.3 Mpa at 365 days. In the work by Ramdane et al. [29], a decrease in compressive strength was observed with increasing WA content. At a 15% WA replacement level and 90 days of curing, the compressive strength reached 30 Mpa, compared to 42 Mpa for the reference mixture. A similar trend was observed for bending strength, where the reference and WA-containing specimens achieved 7.5 Mpa and 5.4 Mpa, respectively.

### 3.6. Total Water Absorption (TWA) and UPV Results

**Figure 8:**
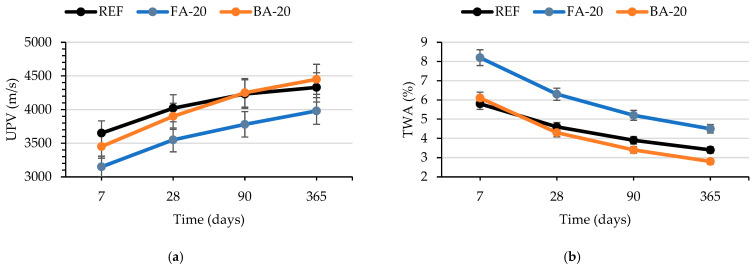
Comparison of (**a**) UPV results; (**b**) TWA results.

The UPV and TWA results, shown in Figure 8, indicate an evolution of the microstructural development in REF, FA-20, and BA-20 over the curing period. At early ages, REF exhibited comparatively higher UPV and lower TWA values up to 28 days, suggesting a more continuous internal structure and a refinement of the pore system, as indicated by the combined UPV and TWA results relative to FA-20 and BA-20. The incorporation of WFA and WBA initially resulted in a less compact microstructure, which can be attributed to cement dilution, leading to increased pore connectivity and reduced ultrasonic wave transmission. These differences may also reflect the combined influence of the WA type and the different preprocessing procedures applied to WFA and WBA, which affected their particle size distribution and, consequently, their packing behavior within the cementitious matrix.

With extended curing, the BA-20 demonstrated a progressive improvement in performance, reflected by an increase in UPV and a reduction in TWA. This behavior suggests gradual microstructural densification over time, associated with continued hydration processes and improved particle packing within the matrix. These changes contribute to pore refinement, enhanced continuity of the solid phase, and a more compact internal structure. This different evolution of the REF and BA-20 systems accounts for the observed inversion in UPV development at later ages. This inversion may be attributed to progressive pore refinement and filler-induced densification in the BA-20 system, which gradually enhances particle packing and improves ultrasonic wave transmission during later curing stages. Consequently, the BA-20 exhibited a denser microstructure at later ages compared to REF. However, the FA-20 consistently exhibited the poorest microstructural development in terms of both UPV and TWA. Some studies have reported that the incorporation of WA up to a replacement level of 20% does not significantly affect water absorption, with values remaining around 5% at 20% replacement [12]. However, other studies have observed an increasing trend in water absorption with higher WA content [30]. The relationship between TWA and UPV is mainly associated with the pore structure and internal compactness of the cementitious matrix. Higher water absorption generally indicates increased porosity and pore connectivity, which can reduce ultrasonic pulse transmission and consequently lower UPV values [31].

The improved performance of BA-20 compared with FA-20 can be explained by its improved particle packing and the evolution of its microstructure, as confirmed by the UPV and TWA results. BA-20 exhibited higher UPV values than FA-20 throughout both early and long-term curing periods (Figure 8a), indicating a more continuous and denser internal structure. This trend is consistent with the TWA results (Figure 8b), where BA-20 showed lower water absorption at all testing ages, suggesting reduced porosity and improved pore connectivity. These microstructural advantages are reflected in the mechanical performance (Figure 7), as BA-20 consistently achieved higher compressive and bending strengths than FA-20. The combined interpretation of Figure 7 and Figure 8 indicates that the better performance of BA-20 is associated with a denser microstructure resulting from improved particle packing and progressive long-term matrix densification.

### 3.7. Alkali-Silica Reaction (ASR) Test

**Figure 9:**
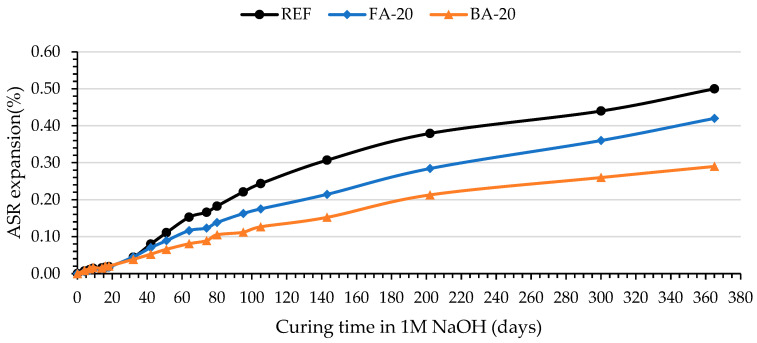
ASR curves of REF, FA-20, and BA-20.

The ASR behavior of the mortar specimens is shown in Figure 9. This test was carried out in an accelerated reaction environment of 1 M NaOH solution at 80 °C. During the first 30 days of exposure, all mixtures exhibited only limited ASR expansion (%), with no significant differences observed between the specimens. Thereafter, the expansion progressively increased, particularly for the REF, which exhibited the highest expansion throughout the test period and reached approximately 0.5% after 365 days. The incorporation of 20% WFA and 20% WBA reduced the expansion to approximately 0.41% and 0.29%, respectively. Both ash-containing mixtures delayed the onset of significant expansion and showed lower expansion than the REF at all curing ages. Among the investigated mixtures, BA-20 was the most effective in mitigating ASR-related expansion, followed by FA-20.

Since the aggregate type and content were identical in all mixtures, the observed differences in ASR expansion are mainly associated with changes in the cementitious phase. The lower expansion observed for the FA-20 and BA-20 may be attributed to the dilution of cement content due to the 20% PC replacement level. Under the accelerated NaOH exposure conditions, this dilution likely modified the characteristics of the cement–WA system and reduced the expansion response compared with the REF. This observation is supported by the findings of Ramos et al. [32], who investigated ASR in hardened mortar specimens in which PC was partially replaced by 10% and 20% WA. Their results indicated that the reference mixture exhibited the highest expansion, while the mixture containing 20% WA showed the lowest expansion, which is consistent with the PC dilution effect.

In addition, the higher expansion observed for the FA-20 compared with the BA-20 may be associated with differences in the mortar pore structure. According to the UPV and TWA tests (Figure 8), the FA-20 developed a more porous and less dense microstructure, which could have facilitated alkali transport under highly alkaline conditions. The ASR behavior may also be influenced by modifications in the C–S–H gel structure, which can affect alkali binding capacity and the chemical environment within the cementitious matrix, thereby contributing to differences in expansion between the investigated mixtures. These combined effects may explain the intermediate expansion behavior observed for the FA-20.

### 3.8. Accelerated Carbonation Tests

**Figure 10:**
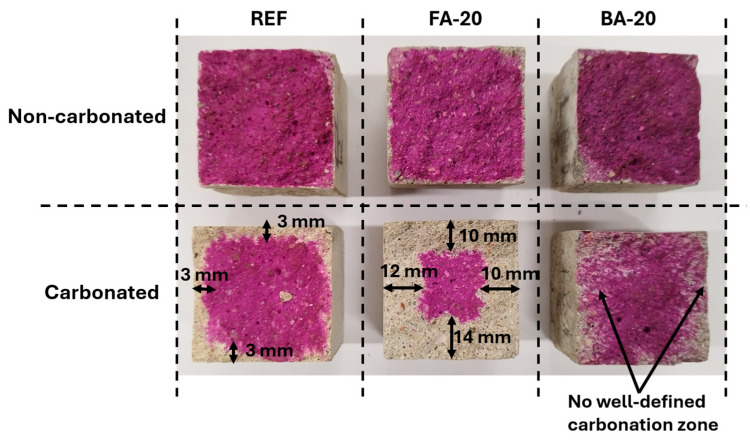
Non-carbonated and carbonated specimens of REF, FA-20, and BA-20.

Figure 10 presents the phenolphthalein test results of the non-carbonated and carbonated mortar specimens after 14 days of accelerated carbonation exposure conducted at a CO_2_ concentration of 20%, temperature of 20 °C, and relative humidity of 70%. The purple coloration indicates non-carbonated alkaline regions, while the uncolored/light regions correspond to carbonated zones.

The REF exhibited the highest resistance to carbonation. Only a shallow carbonation front was observed, with an average carbonation depth of approximately 3 mm from the exposed surfaces. The limited penetration of carbonation suggests a dense microstructure and sufficient alkalinity provided by PC hydration products.

In contrast, the FA-20 showed substantially higher carbonation depths. Localized carbonation penetration of approximately 10–14 mm was measured, indicating a significant reduction in carbonation resistance compared with the REF. This behavior can be attributed to the dilution of cement content, which reduces the amount of calcium hydroxide available to buffer CO_2_ ingress. In addition, the incorporation of WFA may have modified the pore structure, as reflected by lower UPV and higher TWA values, leading to increased permeability and deeper carbonation penetration.

The BA-20 demonstrated a shallow and non-uniform carbonation front, with localized penetration slightly deeper than REF but substantially lower than FA-20. This heterogeneous carbonation pattern indicates the absence of a well-defined carbonation zone and suggests partial and irregular CO_2_ ingress near the exposed surfaces. This suggests that WBA had a less detrimental effect on carbonation resistance than WFA under the investigated conditions. Although all specimens were water-cured for 365 days prior to testing, clear differences in carbonation behavior were still observed between the mixtures. This indicates that the composition of the cement–WA system had a significant influence on long-term carbonation resistance. The results further suggest that replacing PC with WA can increase carbonation susceptibility, particularly in the case of WFA. Beyond the 1-year exposure period investigated in this study, the long-term behavior of BA-20 is expected to be governed by continued microstructural densification due to ongoing hydration and reduced pore connectivity. This may limit CO_2_ ingress and slow carbonation progression; however, reduced portlandite content from PC replacement may decrease the buffering capacity of the system. Therefore, long-term carbonation resistance results from the balance between physical densification and chemical composition, and the effect on reinforcement cannot be directly assessed within this study as it depends on carbonation depth relative to concrete cover. Similar findings were reported by Ramos et al. [32], who investigated WA replacement levels of 10% and 20% as partial replacement of PC. They concluded that carbonation depth increases with increasing WA content, due to the reduction in portlandite and the consequent decrease in pH.

## 4. Conclusions

This study investigated the influence of wood fly ash (WFA) and wood bottom ash (WBA) as partial replacements of Portland cement (PC) on the fresh, mechanical, and durability-related properties of mortar. The results show the effects of ash type and particle characteristics on the long-term performance of the mixtures under mechanical loading and durability-related exposure.

1. The incorporation of WFA and WBA modifies the fresh and hardened behavior of mortar, with both materials influencing microstructural development, mechanical performance, and durability-related properties compared with the REF.
2. UPV results indicated that the reference mortar developed the most rapid and dense internal structure at early ages, while BA-20 showed intermediate development and FA-20 exhibited the most delayed and less dense structural formation.
3. The replacement of PC with WFA and WBA reduced compressive and bending strength compared with the reference mortar, with FA-20 showing the lowest performance and BA-20 exhibiting intermediate strength development.
4. The improved performance of BA-20 relative to FA-20 is associated with differences in particle characteristics, including broader particle size distribution and improved packing, which contributed to a denser mortar structure, as supported by UPV and TWA results.
5. Durability-related tests showed that FA-20 had higher susceptibility to carbonation and ASR expansion, while BA-20 demonstrated improved resistance compared with FA-20, although still lower than the reference mortar. These differences are mainly attributed to Portland cement dilution and resulting changes in microstructure and transport properties.
6. Based on the obtained results, WBA showed more favorable performance than WFA among the properties investigated in this study, due to its comparatively better mechanical performance and improved durability-related behavior. However, additional durability investigations, particularly freeze–thaw resistance testing, are recommended to further assess the durability performance and suitability of wood ash-containing cementitious materials for practical applications.
7. Future studies should consider different replacement levels, including lower substitution rates, together with detailed investigations of microstructural, porosity, and chemical evolution over time, as well as freeze–thaw resistance testing, to further assess the long-term durability performance and applicability of wood ash-containing cementitious systems.

## Figures and Tables

**Table 1:** Chemical composition of PC [17].

**Compound**	SiO_2_	Al_2_O_3_	Fe_2_O_3_	CaO	MgO	SO_3_	K_2_O	Na_2_O	Cl
**Result (%)**	20.9	4.5	4.4	65.2	1.0	2.2	0.7	0.2	0.05

**Table 2:** Mixture compositions of mortars.

Designation	PC (g)	WFA (g)	WBA (g)	Water (g)	Sand (g)	SP (g)
REF	1000	-	-	450	2800	5
FA-20	800	200	-	450	2800	25
BA-20	800	-	200	450	2800	11

**Table 3:** Chemical composition of WFA and WBA.

**Compound**	SiO_2_	CaO	Al_2_O_3_	SO_3_	Fe_2_O_3_	K_2_O	MgO	P_2_O_5_	MnO	Na_2_O	Cl	ZnO
**WFA**	4.2	26.2	1.5	21.4	1.3	23.8	6.4	6.1	0.8	2.2	3.2	2.5
**WBA**	49.6	24.2	5.6	2.8	1.8	6.8	3.4	3.6	0.3	1.2	0.3	0.1

## Data Availability

The original contributions presented in this study are included in the article. Further inquiries can be directed to the corresponding authors.
